# Different Requirements for GFRα2-Signaling in Three Populations of Cutaneous Sensory Neurons

**DOI:** 10.1371/journal.pone.0104764

**Published:** 2014-08-11

**Authors:** Jussi Kupari, Matti S. Airaksinen

**Affiliations:** Institute of Biomedicine, Anatomy, University of Helsinki, Helsinki, Finland; NIH/NEI, United States of America

## Abstract

Many primary sensory neurons in mouse dorsal root ganglia (DRG) express one or several GFRα’s, the ligand-binding receptors of the GDNF family, and their common signaling receptor Ret. GFRα2, the principal receptor for neurturin, is expressed in most of the small nonpeptidergic DRG neurons, but also in some large DRG neurons that start to express Ret earlier. Previously, GFRα2 has been shown to be crucial for the soma size of small nonpeptidergic nociceptors and for their target innervation of glabrous epidermis. However, little is known about this receptor in other Ret-expressing DRG neuron populations. Here we have investigated two populations of Ret-positive low-threshold mechanoreceptors that innervate different types of hair follicles on mouse back skin: the small C-LTMRs and the large Aβ-LTMRs. Using GFRα2-KO mice and immunohistochemistry we found that, similar to the nonpeptidergic nociceptors, GFRα2 controls the cell size but not the survival of both C-LTMRs and Aβ-LTMRs. In contrast to the nonpeptidergic neurons, GFRα2 is not required for the target innervation of C-LTMRs and Aβ-LTMRs in the back skin. These results suggest that different factors drive target innervation in these three populations of neurons. In addition, the observation that the large Ret-positive DRG neurons lack GFRα2 immunoreactivity in mature animals suggests that these neurons switch their GFRα signaling pathways during postnatal development.

## Introduction

The primary somatosensory neurons responsible for conducting different modalities of information from the external world are located outside the CNS in the (spinal) dorsal root ganglia (DRG) and (cranial) trigeminal ganglia. In adult mice, most DRG neurons express the receptor tyrosine kinase Ret, the main signaling receptor for the GDNF family of neurotrophic factors [1]. This family consists of four ligands: GDNF, neurturin (NRTN), artemin, and persephin [2]. They signal through Ret via cognate GFRα receptors, three of which (GFRα1 through GFRα3) are expressed in partially overlapping sensory neuron populations [2]. Of these receptors, GFRα2, the principal receptor for NRTN [3], [4], is the most widely expressed in DRGs [5]. Most Ret^+^/GFRα2^+^ DRG neurons are small, nonpeptidergic primary sensory neurons that extend unmyelinated C-fibers to their targets [1], [5]–[7]. For the most part, these neurons consist of polymodal nociceptors that bind the plant isolectin B4 (IB4) and are known to switch from TrkA to Ret dependency during late embryonic and early postnatal development [1], [5]–[8]. Additionally, a few other populations of small nonpeptidergic Ret^+^ DRG populations have been described [9]–[12]. In contrast to the nonpeptidergic neurons, a population of large Ret^+^/GFRα2^+^ DRG neurons starts to express Ret earlier during development [1], [5], [13]–[16]. These “early-Ret” neurons have been shown to constitute a group of myelinated, rapidly adapting (RA) low threshold mechanoreceptors (Aβ-LTMRs) [15]–[17].

GFRα2/Ret-signaling is known to provide essential trophic signals for proper soma size and epidermal innervation in the IB4-binding nonpeptidergic neurons [5], [7], [18], [19]. However, the importance of GFRα2 mediated signaling in the other nonpeptidergic and in the early-Ret populations of neurons remains poorly known. Here, we have investigated the role of GFRα2-signaling in three different populations of Ret^+^/GFRα2^+^ DRG neurons that innervate the hairy skin: (1) small Mas-related G-protein-coupled receptor D expressing (MrgD^+^) polymodal nociceptors [9], (2) small tyrosine hydroxylase positive (TH^+^) nonpeptidergic C- low threshold mechanoreceptors (TH^+^/C-LTMRs) [11], [12], and (3) large myelinated Ret^+^ neurons (putative Aβ-LTMRs) [15], [16]. We found that GFRα2 is crucial for normal cell size in all the three neuronal populations. Surprisingly, although GFRα2-signaling is required for innervation of hairy skin epidermis by the MrgD^+^ neurons, our results show that it is dispensable for the innervation of hair follicles by the two types of low threshold mechanoreceptors.

## Materials and Methods

### Ethical Statement

The use of animals in this study was in accordance with the relevant EU directive and Finnish legislation (Directive 2010/63/EU, Law 2013/497). The work was authorized by the University of Helsinki Laboratory Animal Center (Internal animal license number: KEK11-002).

### Animals, Tissue Processing and Immunohistochemistry

Adult GFRα2-knockout (KO) [20] and wild type (WT) littermate mice of both sexes in C57BL/6JOlaHsd background were used in this study. In addition, some newborn (P0) and adult *Mrgprd*Δ^EGFPf^ [9] and GFRα2-KO; *Mrgprd*Δ^EGFPf^ animals mice were used.

Mice were anesthetized with an overdose of pentobarbital (200 mg/kg, i.p.; Mebunat Vet, Orion, Finland) and transcardially perfused with 10 ml of cold PBS (pH 7.4), followed by 30 ml of 4% paraformaldehyde (PFA). Lumbar (L4, L5) and thoracic (T5–T8) dorsal root ganglia and sheets of back and hind paw hairy skin were dissected and post-fixed in 4% PFA for 1 hour. Newborn mice were killed by rapid decapitation, and tissue blocks containing the spinal cord and DRGs were immersion-fixed in 4% PFA. The tissues were washed with PBS, cryoprotected in 30% sucrose overnight at +4°C and embedded in Tissue-Tek OCT-compound (Sakura Finetek, USA). For colocalization studies, the DRGs were cut into 16 µm sections. For neuronal number estimation, the DRGs were cut into five series of 10 µm sections, each containing a systematic sample of 1/5 or 1/10 of the full ganglion. Skin samples were cut into 50 µm thick free-floating sections in oblique or longitudinal orientations.

For immunohistochemistry, the DRG sections and free-floating skin sections were blocked in 5% normal donkey serum (NDS) and incubated overnight in a primary antibody solution at room temperature. The sections were then washed twice with PBS and incubated in a secondary antibody solution for 2 hours at room temperature. This was followed by another PBS washing step. The primary antibodies used were against GFRα2, Ret, tyrosine hydroxylase, neurofilament heavy chain (NFH), TrkB, GFP and PGP9.5 (for more specific information, see Table S1). Donkey antibodies from Jackson Immunoresearch with Cy2, Cy3, DyLight488 or DyLight649 fluorophores (diluted 1∶400) were used as secondary antibodies.

### Microscopy and Imaging

A Zeiss Axioplan2 microscope (Carl Zeiss AG, Germany) was used in the investigation of DRG neurons and innervation patterns. Digital images were captured using the Axiovision 4.8 software (Zeiss) and analyzed with ImageJ (NIH). Confocal stacks from the hairy skin were captured with a Leica SP8 laser confocal microscope and conventional microscopy stacks from the DRGs and hairy skin were captured with the Zeiss microscope. The stacks were processed to maximum projections with ImageJ.

### DRG Neuron Numbers, Size Distributions and Marker Colocalization

The numbers of TH^+^ and Ret^+^/NFH^+^ neuronal profiles in the DRGs were estimated by staining every fifth (TH) or tenth (Ret/NFH) section from each DRG and counting the number of positive cell profiles with a nucleus. Because of the neuronal size difference between the genotypes, the results were corrected using the method described by Abercrombie [21]. Briefly, the corrected estimate (*N*) for each section was calculated with the equation: *N = (n×T)÷(T+D),* where *n* is the number of counted profiles, *T* is the thickness of the section and *D* is the average profile diameter. In this case, *D* was calculated from the mean neuronal profile area. Digital images were captured at 200X magnification, and neuronal profile size distributions were determined from the digital images by measuring the areas from positive, nucleus-containing cell profiles. At least three animals per genotype and two DRGs per animal were used in the analysis of cell numbers and size distributions. Colocalization was analyzed from at least two animals per genotype and two DRGs per animal.

### Quantification of Hair Follicle and Back Skin Epidermal Innervation

The relative portion of hair follicles with visible TH^+^ longitudinal lanceolate endings (LLEs) was quantified as follows: using bright-field optics (10x and 20x objectives), the section was carefully scanned for small caliber hair follicles with visible sebaceous glands (Fig. S1A). When found, the presence of LLEs was investigated by switching to fluorescence imaging (20x and 40x objectives). Follicles with clearly visible LLEs were counted as positive and follicles without LLEs were marked as negative. In the case of NFH^+^ LLEs, the sections were carefully scanned (5x and 10x objectives) for large, singular hair follicles (Fig. S1B). Once found, the fluorescence analysis was done as for TH^+^ endings. For both TH^+^ and NFH^+^ endings, at least 60 hair follicles per animal were analyzed. The density of EGFP-positive (MrgD^+^) epidermal free nerve endings in the back skin and dorsal paw hairy skin were analyzed from randomly selected optical fields from sections stained for EGFP. Using a 40x objective, the whole thickness of the section was carefully scanned through, and all positive nerve fibers crossing the dermis-epidermis border were counted. The length of the border in the optical field was measured and the innervation was represented as the number of nerve fiber crossings per length of border.

### Statistics

Differences between genotypes were compared by using the Mann-Whitney U-test (two-tailed) for average neuron numbers, and average innervated hair follicles. Neuronal size distributions were analyzed using the chi-square test. The statistical analyses were done using R (www.r-project.org/). In the case of neuronal size distributions, some bins were merged to reach the required frequency of five or more in at least 80% of the bins. The threshold for statistical significance was set at p<0.05. The results are presented as mean ± SEM.

## Results

### Reduced MrgD^+^ Neuron Size and Deficient Epidermal Innervation in GFRα2-KO Hairy Skin

**Figure 1:**
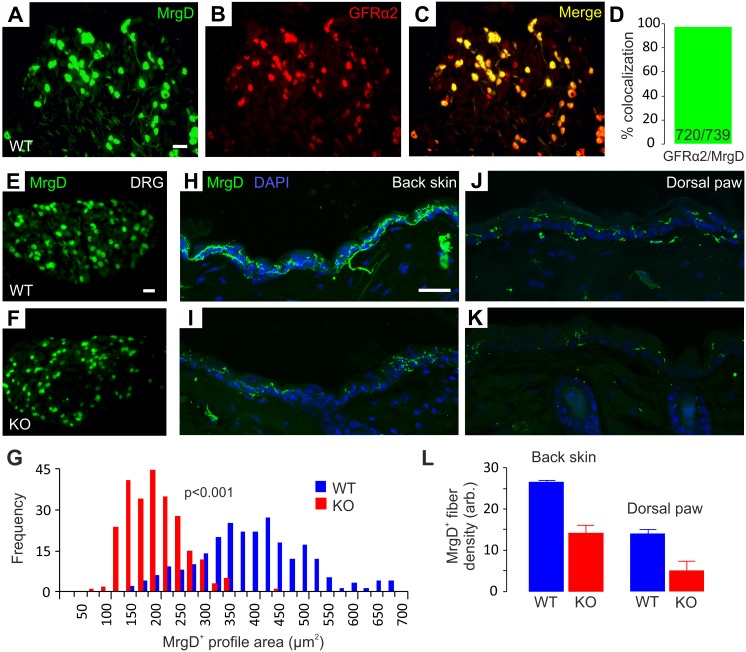
MrgD^+^ DRG neurons in GFRα2-KO mice are smaller and deficient in epidermal innervation of hairy skin. (A–C) Extensive colocalization of EGFP and GFRα2 in *Mrgprd*Δ^EGFPf^ mouse DRGs. (D) Virtually all MrgD^+^ neurons express GFRα2 (GFRα2^+^ cells/total MrgD^+^ cells, n = 4 animals). (E-G) MrgD^+^ DRG neurons are smaller in GFRα2-KO than in wild-type animals (G: WT 382±34 µm^2^, KO 202±15 µm^2^; the data are from 270 cells and three animals per genotype, chi-square -test). (H–L) MrgD^+^ epidermal innervation is denser in the hairy skin of wild-type than GFRα2-KO mice (L: back skin, WT 26.8±0.4, KO 14.5±2.0; dorsal paw, WT 14.2±1.0, KO 5.1±2.3 arbitrary units; the data are from two animals/genotype and 2–4 skin samples/animal). Scale bars: (A, E) 50 µm; (H) 20 µm.

GFRα2-signaling is crucial for proper IB4^+^ nonpeptidergic neuron size and innervation of the glabrous epidermis [7]. To investigate if this phenotype extends to hairy skin, we used mice in which the *Mrgprd* gene locus had been targeted with an EGFP expressing construct [9]. This gene encodes MrgD, a Mas-related G-protein coupled receptor specifically expressed in nonpeptidergic nociceptive neurons that represent a great majority (75%) of the IB4^+^ DRG neurons [9]. As expected, nearly all (>97%) EGFP-positive ( = MrgD^+^) neurons were positive for GFRα2 in wild-type (WT) DRGs (Fig. 1A–D). We also analyzed the size distribution of these cells and found that the MrgD^+^ neurons were drastically smaller in KO mice than in WT animals (Fig. 1E, F, G).

Next, we studied the innervation density of MrgD^+^ fibers in the back and hind paw hairy skin. MrgD^+^ fibers were abundant in WT back skin epidermis and slightly sparser in the dorsal hind paw (Fig. 1H, J, L). Compatible with the phenotype found on GFRα2-KO glabrous epidermis [7], the KO animals showed a reduced density (50–70% loss) of MrgD^+^ innervation on both back and dorsal paw epidermis (Fig. 1I, K, L). A qualitatively similar difference in back skin innervation was also observed using the pan-neuronal PGP9.5 immunostaining in additional WT and KO animals (Fig. S2).

### TH^+^ and Ret^+^/NFH^+^ Neurons Survive But Are Smaller in GFRα2-KO Thoracic DRGs

**Figure 2:**
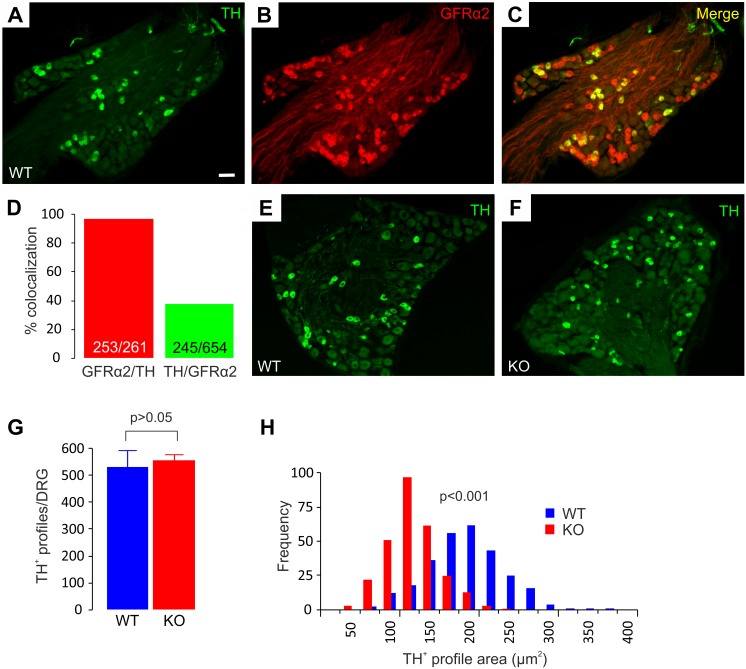
TH^+^ DRG neurons in GFRα2-KO mice are smaller. (A–C) TH and GFRα2 colocalize in a subpopulation of thoracic DRG neurons. (D) Nearly all TH^+^ neurons express GFRα2 (red bar). TH^+^ neurons comprise ∼37% of all GFRα2 neurons (green bar). Analysis was done from three animals and three ganglia in total. (E–H) The number of TH^+^ neurons in thoracic DRGs is similar between the genotypes (G: WT 533±59, KO 555±24; positive neurons per DRG, n = 3 animals/genotype, U-test) but the size is smaller in the knock-out animals (H: WT 196±14 µm^2^, KO 120±8 µm^2^; three animals and 200 cells/genotype in total, chi-square -test). Scale bar: 50 µm.

A distinct class of C-LTMRs that expresses vesicular glutamate transporter VGlut3 [11] and tyrosine hydroxylase (TH) [12] constitutes a subpopulation of nonpeptidergic C-fibers. This population of neurons has also been reported to express GFRα2 and Ret, but not to bind IB4 or express any of the Mas-related G-protein receptors, making them a separate population from the nonpeptidergic nociceptors [12]. Using immunohistochemistry and two different antibodies, we confirmed that a significant population of thoracic DRG neurons expresses TH in wild-type mice (Fig. 2A, E and Fig. S4). Co-staining showed that nearly all of the TH^+^ DRG neurons expressed GFRα2 (Fig. 2B–D). Vice versa, the TH^+^ neurons totaled to a roughly one third of all GFRα2^+^ neurons (Fig. 2D). To investigate if GFRα2 is required for TH expression or survival of these DRG neurons, we quantified the numbers of TH^+^ neurons in thoracic DRGs of both wild-type and GFRα2-KO animals. Firstly, intense TH^+^ immunoreactivity was found on a subpopulation of neurons in KO DRGs, indicating that GFRα2-signaling is not a requirement for TH expression (Fig. 2F). Secondly, no significant differences were found in the number estimates of TH^+^ neurons per DRG between the genotypes, suggesting that GFRα2 is not required for TH^+^ neuron survival (Fig. 2G). We then performed a cell size distribution analysis, which showed that the TH^+^ neurons were significantly smaller in the GFRα2-KO animals (Fig. 2H).

**Figure 3:**
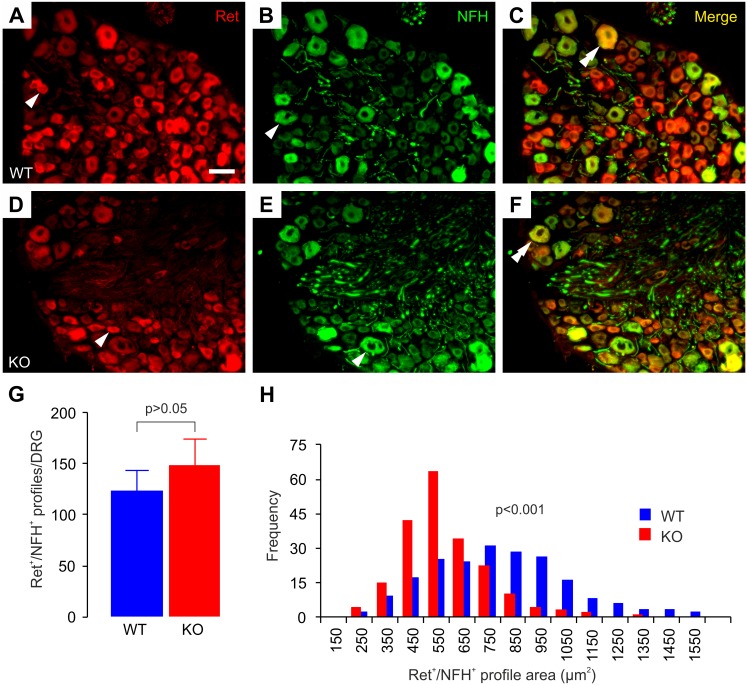
Ret^+^/NFH^+^ DRG neurons in GFRα2-KO mice are also smaller. (A–F) Ret and NFH colocalize in a population of thoracic DRG neurons in both wild-type and GFRα2-KO mice. Single arrowheads in (A, B, D, E) point to examples of single-positive neurons. Double arrowheads in (C, F) point to examples of double positive neurons. (G) GFRα2-KO thoracic DRGs have normal numbers of Ret^+^/NFH^+^ neurons (WT 124±21 (n = 4 animals), KO 149±26 (n = 3 animals); double-positive neurons per ganglion, U-test). (H) The size distribution of Ret^+^/NFH^+^ thoracic DRG neurons has shifted toward smaller sizes in KO animals (WT 757±21, KO 557±12 µm^2^; three animals/group, 250 cells per genotype, chi-square -test). Scale bar: 50 µm.

In contrast to the small nonpeptidergic nociceptors and mechanoreceptors that switch from NGF- to Ret-dependency during late prenatal and early postnatal life, the large early-Ret population of DRG neurons expresses Ret and GFRα2 from early embryonic stages [15], [16]. In accordance with this, we found GFRα2-immunoreactivity only in few large DRG neurons at P0 (Fig. S3A). These neurons were clearly a separate population from the MrgD^+^ neurons that make up the bulk of nonpeptidergic DRG neurons (Fig. S3B, C). Surprisingly, we were unable to detect GFRα2-immunoreativity in adult DRG NFH^+^ neurons (Fig. S3D–F). Since the early-Ret neurons express NFH in addition to Ret [15], [16], we used antibodies against these two markers to investigate this neuronal population in thoracic DRGs. Most Ret^+^ neurons in adult mouse thoracic DRGs are small and NFH-negative (Fig. 3A, D; arrowheads), and many large NFH^+^ neurons are negative for Ret (Fig. 3B, E; arrowheads). In addition, we observed a population of large Ret^+^/NFH^+^ cells in both WT and GFRα2-KO mice (Fig. 3C, F; double arrowheads). Quantification of the numbers of these double-positive neurons revealed no significant differences between the genotypes (Fig. 3G), but the cell size distributions indicated that the average size of these neurons was clearly smaller in the KO than in WT animals (Fig. 3H).

### GFRα2-KO Mice Have a Normal Pattern of Hair Follicle Innervation

**Figure 4:**
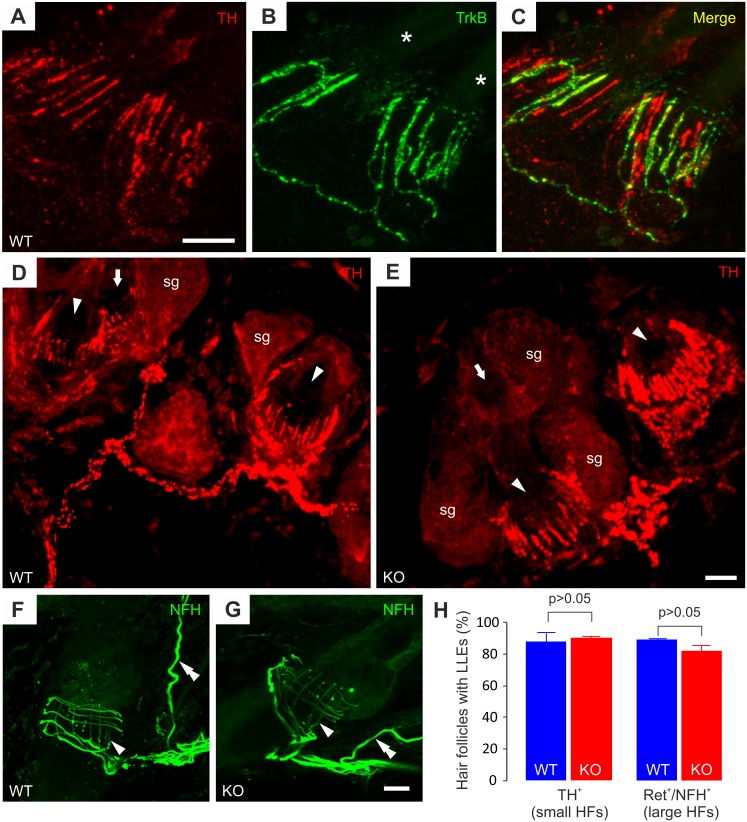
C- and Aβ-LTMR innervation of hair follicles in GFRα2-KO mouse back skin remains unchanged. (A–C) The images show two small caliber hair follicles with TH^+^ and TrkB^+^ LLEs. Immunoreactivity for the markers is localized in different populations of endings (C). The asterisks in (B) mark the two separate hair shafts. (D, E) The images demonstrate TH^+^ sensory innervation around small caliber hair follicles in wild-type (D) and KO (E) back skin. The arrowheads indicate obliquely cut hair follicles with TH^+^ LLEs. The small arrows mark hair follicles with few or no visible endings. Sebaceous glands (sg) around the follicles are indicated. No changes are obvious in the morphology of TH^+^ LLEs around GFRα2-KO hair follicles. (I, left side) The percentage of small hair follicles that are innervated by TH^+^ LLEs is similar between the genotypes (WT 87.8±6.4%, KO 90.6±1.1%; three animals/genotype, 60–80 small hair follicles/animal, U-test). (F, G) The morphology of NFH^+^ LLEs (arrowheads) appears similar between the genotypes. Double arrowheads point to nerve structures innervating the touch dome (located outside of the image). (I, right side) NFH^+^ LLEs remain in KO back skin (WT 88.7±0.8%, KO 81.6±3.6%; innervated follicles/total follicles, three animals/genotype, 60–80 small hair follicles/animal, U-test). Scale bars: 10 µm.

To see if the diminished sizes of TH^+^ and Ret^+^/NFH^+^ neurons in GFRα2-KO mice reflected in changes of their peripheral nerve processes, we studied their end-organ innervation patterns in the hairy skin. The TH^+^/C-LTMRs have been shown to exclusively innervate the smaller types of hair follicles on mouse skin, forming LLEs around the outer root sheath [12], [22]. These studies have used mice with a genetic TH-reporter to visualize the C-LTMR innervation. Since the cell bodies of TH^+^/C-LTMRs were readily visible with TH-antibodies, we examined if also the sensory TH^+^ fibers could be visualized by immunohistochemistry. The LLE complex is located between the origin of the *arrector pili* muscle on the hair follicle and the sebaceous gland associated with the follicle [23]. We used these landmarks to localize possible TH^+^ sensory endings on hair follicles. Indeed, TH^+^ LLEs were visible around the smaller hair follicles, but with only one of the two antibodies (rabbit anti-TH) (Fig. 4A). Because these follicles are also innervated by LLEs from another type of LTMRs, the Aδ-LTMRs, we used TrkB (a marker for the Aδ-LTMRs) [12], [17] as a control for the specificity of our TH-antibody. Double-staining against TH and TrkB showed that these two markers were expressed in two separate populations of LLEs, demonstrating that our TH-antibody stains specifically C-LTMRs on the hair follicles (Fig. 4A–C).

Due to the similar phenotypic changes in TH^+^ and MrgD^+^ neuron somas in GFRα2-KO animals, we hypothesized that the density of TH^+^/C-LTMR innervation around hair follicles could also be reduced in the KO mice. Surprisingly, we found an apparently similar pattern and morphology of TH^+^/C-LTMR innervation in WT and KO skin (Figure 4 D, E). A thorough quantification indicated no difference in the proportion of hair follicles innervated by TH^+^ fibers between the genotypes (Fig. 4H, left).

Lastly, we proceeded to investigate hairy skin innervation by the Ret^+^/NFH^+^ DRG neurons. In the back skin the RA Aβ-LTMRs form LLEs around the larger awl/auchene and guard types of hair follicles [15]. In concurrence with this, we observed NFH^+^ longitudinal and circular endings around most large hair follicles in WT back skin. In the smaller hair follicles, only circular NFH^+^ endings were visible (not shown). A previous study has suggested that these RA Aβ-LTMRs are critically dependent on Ret-signaling for target innervation [16]. As these neurons express GFRα2 during development, we expected to see a lacking phenotype in the GFRα2-KO mice. However, NFH^+^ endings were present around large hair follicles in the GFRα2-KO mice (Fig. 4G), and quantification of the percentage of large hair follicles with NFH^+^ LLEs indicated no difference between the WT and GFRα2-KO animals (Fig. 4H, right). Also, the morphology of NFH^+^ LLEs and circular endings appeared similar between the genotypes (Fig. 4F, G).

## Discussion

The key finding of the present study is that GFRα2 is required for proper size of both TH^+^/C-LTMR and RA Aβ-LTMR neurons, but is expendable for their target innervation of hair follicles, in contrast to the MrgD^+^ neurons that depend on GFRα2 for both cell size and target innervation of the epidermis.

### MrgD^+^ and TH^+^/C-LTMRs Require GFRα2-Signaling for Size But Not for Survival

We found that both MrgD^+^ and TH^+^ DRG neurons are clearly undersized in the GFRα2-KO animals. These results support the previous conclusion that GFRα2-signaling is an important factor in controlling the soma size of nonpeptidergic sensory neurons: over 80% of IB4^+^ DRG neurons express GFRα2, and these cells are visibly smaller in GFRα2-KO mice [7]. This phenotype of diminished cell size has also been shown in peripherin expressing DRG neurons [5], Ret^+^ nociceptors [19] and Ret^+^/IB4^+^ and Ret^+^/IB4^−^ neurons [18] in different Ret conditional knock-out (cKO) mouse models. The importance of neurturin on nonpeptidergic DRG neurons is also evident in mice that over-express this ligand in keratinocytes, as in these animals the size and percentage of GFRα2^+^ DRG neurons is radically increased [24]. Consistent with our previous observations that GFRα2-KO mice have normal numbers of small IB4^+^/P2X_3_^+^ DRG neurons [7] and unmyelinated axons in cutaneous nerves [25], we found no decrease in the numbers of TH^+^ neurons in GFRα2-KO thoracic DRGs, reinforcing the view that GFRα2-signaling is not required for survival in nonpeptidergic DRG neurons. The MrgD^+^ neurons also appeared to be present in normal numbers in KO DRGs, in line with the fact that a virtually all MrgD^+^ neurons bind IB4 and coexpress the ATP-gated channel P2X_3_ [9]. Furthermore, most reports indicate that Ret-signaling is not essential for the in vivo survival of nonpeptidergic DRG neurons [5], [18], [26] (however, see [19]).

### TH^+^/C-LTMRs Do Not Require GFRα2 for Target Innervation

We found that the density of MrgD^+^ fibers is reduced in GFRα2-KO back skin epidermis, consistent with the scarcity of nonpeptidergic innervation in the glabrous skin of these mice [7] and in both glabrous and hairy skin of Ret cKO mice [5], [18]. In striking contrast, the percentage of TH^+^ innervated hair follicles and the morphology of TH^+^ LLEs in the GFRα2-KO mice were similar with WT mice, indicating that target innervation by TH^+^/C-LTMRs is independent of GFRα2-signaling. In adult mice, TH^+^/C-LTMRs do not express other Ret co-receptors in addition to GFRα2 [5], but whether the formation of these endings requires Ret or neurturin has not been reported. In addition to neurotrophic factors, many other repulsive or attractive molecules influence the innervation patterns of nerve fibers [27]. For example, Nogo receptor 2 (NgR2) [28] is expressed on the IB4-binding nonpeptidergic neurons and regulates their epidermal innervation by interacting with the repulsive proteoglycan, versican [29]. Also, neurturin has been shown to prevent axon growth cone collapse caused by another repulsive molecule, semaphorin 3A [30]. These findings suggest that the axon guidance effects of neurotrophic factors could be partially dependent on inhibiting repulsive cues. Since the MrgD^+^ neurons terminate as free nerve endings in the epidermis, whereas the TH^+^/C-LTMR endings terminate just below the epidermis, “anti-repulsive” signaling through GFRα2 is one possible explanation for the different dependence of these endings on GFRα2.

### Anti-TH Antibodies as C-LTMR Markers

We have used here antibodies against TH as a marker for the C-LTMR neurons and their target innervation around hair follicles, whereas the previous studies used genetic labeling of TH- or VGlut3-expressing neurons to visualize the C-LTMR endings in hairy skin [12], [31]. In our hands, two different anti-TH antibodies labeled an identical subpopulation of DRG neurons (Fig. S4) but only one of them, namely the rabbit anti-TH antibody (Table S1), labeled the LLEs (although both antibodies labeled sympathetic nerve fibers) in the skin. The reason for this difference is presently unclear, but this antibody should be a valuable tool for further studies on these neurons in different species.

### Large Ret^+^ Neurons Require GFRα2 for Proper Soma Size Development But Downregulate GFRα2-Expression after Adolescence

Our results show that Ret^+^/NFH^+^ DRG neurons have diminished in size in GFRα2-KO mice. This is in line with the observation that myelinated axons in the saphenous nerves are thinner in GFRα2-KO than in WT mice [25]. Furthermore, in NRTN-overexpressing mice, the largest myelinated fibers in the saphenous nerves are selectively hypertrophied, and these fibers likely correspond to the Ret^+^/NFH^+^ neurons [24]. Collectively these findings indicate that NRTN/GFRα2-signaling plays a significant role in the regulation of cell body and axon size in the Ret^+^ myelinated DRG neurons.

Ret mRNA expression in the mouse DRG starts already during the second week of embryonic development [32]. These large, early-Ret neurons express NFH from P0 onwards [16] and have been shown to express GFRα2 in the mouse [15], [16], [26] at least until P14 [15]. Consistent with this, we saw GFRα2^+^ immunoreactivity at P0 only in large DRG neurons that did not express the nonpeptidergic marker MrgD, in agreement with the view that GFRα2 is expressed only in the early-Ret neurons at this point in development [15]. Interestingly, we saw no immunoreactivity against GFRα2 in the large NFH^+^ DRG neurons in adults, suggesting that GFRα2 is downregulated in the Ret^+^/NFH^+^ neurons after P14 in mice. This also suggests that the large, early-Ret neurons depend on GFRα2 for their size during development but not in adults.

Our observation that the large Ret^+^/NFH^+^ neurons survive in GFRα2-KO supports the earlier finding that the number of myelinated axons in the saphenous nerve of these mice is normal [25]. Furthermore, both in neurturin-KO and in another GFRα2-KO mouse, the large Ret^+^ DRG neurons appear intact at P0 [15]. However, the dependency of these large neurons on Ret-signaling for survival is somewhat unclear. Normal numbers of DRG neurons were reported in one Ret cKO mouse line at P14 [5], but in another Ret cKO mouse model, the number of NFH^+^ DRG neurons was reported to be reduced by 15% at P15.5 [16]. Also, in a Ret^GFP/GFP^ KO mouse line, about one-third of GFP^+^ DRG neurons were reported lost at E14.5 [26]. Consequently, it remains possible that Ret is required for the survival of a subset of myelinated Ret^+^ DRG neurons.

### Formation of Aβ-LTMR Longitudinal Lanceolate Endings Is Independent of GFRα2-Signaling

Our finding that NFH^+^ LLEs are normal in GFRα2-KO mice suggests that signaling mediated through GFRα2 is not essential for target innervation by this subpopulation of RA Aβ-LTMR neurons. On the other hand, Pacinian corpuscles, the end organs of another subset of RA Aβ-LTMR neurons, were reported to be almost completely lost in NRTN-KO mice and in another GFRα2-deficent mouse line [15]. Furthermore, the formation of these two RA Aβ-LTMR end organ types appears to be critically dependent on Ret-signaling, as NFH^+^ LLEs [16] and Pacinian corpuscles [15] are compromised in different Ret cKO models. These results imply that subpopulations of early-Ret neurons differ in their GFRα requirements for target innervation. In addition to our finding that GFRα2-immunoreactivity is not detectable in the large NFH^+^/Ret^+^ neurons, other studies on both mice and rats have indicated that GFRα1 but not GFRα2 (or GFRα3) is expressed in the largest Ret^+^ DRG neurons in adult animals [33], [34]. Together, these results suggest that the early-Ret neurons might switch from GFRα2 to GFRα1 signaling during postnatal maturation.

## Conclusions

We show that GFRα2 is critical for controlling the neuron size (but not survival) of three different subpopulations of DRG neurons in mice: small MrgD^+^ nociceptors, small TH^+^/C-LTMRs and large (Ret^+^/NFH^+^) Aβ-LTMRs. However, in contrast to the MrgD^+^ neurons that require GFRα2 to innervate the epidermis, GFRα2 is not required for the innervation of hair follicles by the two populations of LTMRs. Thus, factors other than GFRα2 drive target innervation in these neurons.

## Supporting Information

Figure S1 **Bright-field images from wild-type mouse back skin.** (A) The image shows several small caliber hair follicles representative of the type used in the analysis of TH^+^ LLE innervation. The black arrowhead points to a sebaceous gland next to a hair follicle. Note that the small hair follicles generally form closely associated pairs (asterisks). (B) The image shows several small and one large caliber hair follicle. The large hair follicle (asterisk) is representative of the type used in NFH^+^ LLE innervation analysis. Note the considerably larger diameter and singular hair shaft (yellow arrows) in the large follicle compared to a small hair follicle (green arrows). Scale bars: 100 µm.
(TIF)

Figure S2 **Epidermal innervation in GFRα2-KO mouse back skin is reduced.** (A) WT mouse back skin epidermis is densely innervated by PGP9.5^+^ nerve fibers. (B) Epidermal innervation is sparse in KO back skin epidermis. Arrowheads in (A) and (B) exemplify the difference in innervation density between the genotypes. Asterisks mark large caliber hair shafts. The images are maximum projections from conventional microscopic stacks. Scale bar: 25 µm.
(TIF)

Figure S3 **GFRα2 is not detectable in large NFH^+^ DRG neurons in adult mice.** (A–C) Conventional microscopic images demonstrating that GFRα2 expression is restricted to a minor population of large DRG neurons at P0 (A), while MrgD is expressed in a major population of smaller neurons (B). (C) GFRα2 and MrgD do not colocalize in newborn mouse DRGs (arrowheads point to single-positive GFRα2^+^ neurons). (D–F) Maximal projection images showing that GFRα2 is abundantly expressed in small to medium diameter neurons in adult mouse thoracic DRGs (D) and that NFH is expressed in many medium to large sized DRG neurons (E). (F) GFRα2 and NFH do not colocalize in adult mouse DRGs. Scale bars: 50 µm.
(TIF)

Figure S4 **Two different anti-TH antibodies stain the same population of DRG neurons.** (A, B) Representative images from a WT mouse thoracic DRG section stained with (A) rabbit anti-TH and (B) sheep anti-TH antibody. (C) The merged image shows that the two antibodies label the same cell population. This virtually 100% colocalization was confirmed by analysis of 356 TH^+^ cells (in five DRGs from two animals). Scale bar: 50 µm.
(TIF)

Table S1 **Primary antibodies used in immunohistochemistry.**
(PDF)

## References

[1] Molliver DC, Wright DE, Leitner ML, Parsadanian AS, Doster K, et al (1997) IB4-Binding DRG Neurons Switch from NGF to GDNF Dependence in Early Postnatal Life. Neuron 19: 849–861 10.1016/S0896-6273(00)80966-6 9354331

[2] Airaksinen MS, Saarma M (2002) The GDNF family: Signalling, biological functions and therapeutic value. Nat Rev Neurosci 3: 383–394 10.1038/nrn812 11988777

[3] Klein RD, Sherman D, Ho W-H, Stone D, Bennett GL, et al (1997) A GPI-linked protein that interacts with Ret to form a candidate neurturin receptor. Nature 387: 717–721 10.1038/42722 9192898

[4] Buj-Bello A, Adu J, Piñón LGP, Horton A, Thompson J, et al (1997) Neurturin responsiveness requires a GPI-linked receptor and the Ret receptor tyrosine kinase. Nature 387: 721–724 10.1038/42729 9192899

[5] Luo W, Wickramasinghe SR, Savitt JM, Griffin JW, Dawson TM, et al (2007) A Hierarchical NGF Signaling Cascade Controls Ret-Dependent and Ret-Independent Events during Development of Nonpeptidergic DRG Neurons. Neuron 54: 739–754 10.1016/j.neuron.2007.04.027 17553423

[6] Bennett DLH, Michael GJ, Ramachandran N, Munson JB, Averill S, et al (1998) A Distinct Subgroup of Small DRG Cells Express GDNF Receptor Components and GDNF Is Protective for These Neurons after Nerve Injury. J Neurosci 18: 3059–3072. 9526023 10.1523/JNEUROSCI.18-08-03059.1998 PMC6792585

[7] Lindfors PH, Võikar V, Rossi J, Airaksinen MS (2006) Deficient Nonpeptidergic Epidermis Innervation and Reduced Inflammatory Pain in Glial Cell Line-Derived Neurotrophic Factor Family Receptor α2 Knock-Out Mice. J Neurosci 26: 1953–1960 10.1523/JNEUROSCI.4065-05.2006 16481427 PMC6674922

[8] Plenderleith MB, Snow PJ (1993) The plant lectin Bandeiraea simplicifolia I-B4 identifies a subpopulation of small diameter primary sensory neurones which innervate the skin in the rat. Neurosci Lett 159: 17–20. 8264961 10.1016/0304-3940(93)90787-l

[9] Zylka MJ, Rice FL, Anderson DJ (2005) Topographically Distinct Epidermal Nociceptive Circuits Revealed by Axonal Tracers Targeted to Mrgprd. Neuron 45: 17–25 10.1016/j.neuron.2004.12.015 15629699

[10] Liu Q, Vrontou S, Rice FL, Zylka MJ, Dong X, et al (2007) Molecular genetic visualization of a rare subset of unmyelinated sensory neurons that may detect gentle touch. Nat Neurosci 10: 946–948 10.1038/nn1937 17618277

[11] Seal RP, Wang X, Guan Y, Raja SN, Woodbury CJ, et al (2009) Injury-induced mechanical hypersensitivity requires C-low threshold mechanoreceptors. Nature 462: 651–655 10.1038/nature08505 19915548 PMC2810205

[12] Li L, Rutlin M, Abraira VE, Cassidy C, Kus L, et al (2011) The Functional Organization of Cutaneous Low-Threshold Mechanosensory Neurons. Cell 147: 1615–1627 10.1016/j.cell.2011.11.027 22196735 PMC3262167

[13] Kramer I, Sigrist M, de Nooij JC, Taniuchi I, Jessell TM, et al (2006) A Role for Runx Transcription Factor Signaling in Dorsal Root Ganglion Sensory Neuron Diversification. Neuron 49: 379–393 10.1016/j.neuron.2006.01.008 16446142

[14] Chen C-L, Broom DC, Liu Y, de Nooij JC, Li Z, et al (2006) Runx1 Determines Nociceptive Sensory Neuron Phenotype and Is Required for Thermal and Neuropathic Pain. Neuron 49: 365–377 10.1016/j.neuron.2005.10.036 16446141

[15] Luo W, Enomoto H, Rice FL, Milbrandt J, Ginty DD (2009) Molecular Identification of Rapidly Adapting Mechanoreceptors and Their Developmental Dependence on Ret Signaling. Neuron 64: 841–856 10.1016/j.neuron.2009.11.003 20064391 PMC2813518

[16] Bourane S, Garces A, Venteo S, Pattyn A, Hubert T, et al (2009) Low-Threshold Mechanoreceptor Subtypes Selectively Express MafA and Are Specified by Ret Signaling. Neuron 64: 857–870 10.1016/j.neuron.2009.12.004 20064392

[17] Wende H, Lechner SG, Cheret C, Bourane S, Kolanczyk ME, et al (2012) The Transcription Factor c-Maf Controls Touch Receptor Development and Function. Science 335: 1373–1376 10.1126/science.1214314 22345400

[18] Franck MCM, Stenqvist A, Li L, Hao J, Usoskin D, et al (2011) Essential role of Ret for defining non-peptidergic nociceptor phenotypes and functions in the adult mouse. Eur J Neurosci 33: 1385–1400 10.1111/j.1460-9568.2011.07634.x 21395865

[19] Golden JP, Hoshi M, Nassar MA, Enomoto H, Wood JN, et al (2010) RET Signaling Is Required for Survival and Normal Function of Nonpeptidergic Nociceptors. J Neurosci 30: 3983–3994 10.1523/JNEUROSCI.5930-09.2010 20237269 PMC2850282

[20] Rossi J, Luukko K, Poteryaev D, Laurikainen A, Sun YF, et al (1999) Retarded growth and deficits in the enteric and parasympathetic nervous system in mice lacking GFR alpha2, a functional neurturin receptor. Neuron 22: 243–252. 10069331 10.1016/s0896-6273(00)81086-7

[21] Abercrombie M (1946) Estimation of nuclear population from microtome sections. Anat Rec 94: 239–247 10.1002/ar.1090940210 21015608

[22] Li L, Ginty DD (2014) The structure and organization of lanceolate mechanosensory complexes at mouse hair follicles. eLife 3: e01901 10.7554/eLife.01901 24569481 PMC3930909

[23] Halata Z (1993) Sensory Innervation of the Hairy Skin (Light- and Electronmicroscopic Study). J Invest Dermatol 101: 75S–81S 10.1111/1523-1747.ep12362877 8326156

[24] Wang T, Jing X, DeBerry JJ, Schwartz ES, Molliver DC, et al (2013) Neurturin Overexpression in Skin Enhances Expression of TRPM8 in Cutaneous Sensory Neurons and Leads to Behavioral Sensitivity to Cool and Menthol. J Neurosci 33: 2060–2070 10.1523/JNEUROSCI.4012-12.2013 23365243 PMC3753026

[25] Stucky CL, Rossi J, Airaksinen MS, Lewin GR (2002) GFR α2/neurturin signalling regulates noxious heat transduction in isolectin B4-binding mouse sensory neurons. J Physiol 545: 43–50 10.1113/jphysiol.2002.027656 12433948 PMC2290664

[26] Honma Y, Kawano M, Kohsaka S, Ogawa M (2010) Axonal projections of mechanoreceptive dorsal root ganglion neurons depend on Ret. Development 137: 2319–2328 10.1242/dev.046995 20534675

[27] Guan K-L, Rao Y (2003) Signalling mechanisms mediating neuronal responses to guidance cues. Nat Rev Neurosci 4: 941–956 10.1038/nrn1254 14682358

[28] Laurén J, Airaksinen MS, Saarma M, Timmusk T (2003) Two novel mammalian nogo receptor homologs differentially expressed in the central and peripheral nervous systems☆. Mol Cell Neurosci 24: 581–594 10.1016/S1044-7431(03)00199-4 14664809

[29] Bäumer BE, Kurz A, Borrie SC, Sickinger S, Dours-Zimmermann MT, et al (2014) Nogo Receptor Homolog NgR2 Expressed in Sensory DRG Neurons Controls Epidermal Innervation by Interaction with Versican. J Neurosci 34: 1633–1646 10.1523/JNEUROSCI.3094-13.2014 24478347 PMC3905139

[30] Wanigasekara Y, Keast JR (2006) Nerve growth factor, glial cell line–derived neurotrophic factor and neurturin prevent semaphorin 3A–mediated growth cone collapse in adult sensory neurons. Neuroscience 142: 369–379 10.1016/j.neuroscience.2006.06.031 16876331

[31] Lou S, Duan B, Vong L, Lowell BB, Ma Q (2013) Runx1 Controls Terminal Morphology and Mechanosensitivity of VGLUT3-expressing C-Mechanoreceptors. J Neurosci 33: 870–882 10.1523/JNEUROSCI.3942-12.2013 23325226 PMC3652638

[32] Pachnis V, Mankoo B, Costantini F (1993) Expression of the c-ret proto-oncogene during mouse embryogenesis. Development 119: 1005–1017. 8306871 10.1242/dev.119.4.1005

[33] Bennett DLH, Boucher TJ, Armanini MP, Poulsen KT, Michael GJ, et al (2000) The Glial Cell Line-Derived Neurotrophic Factor Family Receptor Components Are Differentially Regulated within Sensory Neurons after Nerve Injury. J Neurosci 20: 427–437. 10627618 10.1523/JNEUROSCI.20-01-00427.2000 PMC6774134

[34] Wang T, Molliver DC, Jing X, Schwartz ES, Yang F-C, et al (2011) Phenotypic Switching of Nonpeptidergic Cutaneous Sensory Neurons following Peripheral Nerve Injury. PLoS ONE 6: e28908 10.1371/journal.pone.0028908 22216140 PMC3244441

