# Supplementary figures and images for: Different Requirements for GFRα2-Signaling in Three Populations of Cutaneous Sensory Neurons
Source: PLoS One. 2014 Aug 11;9(8):e104764. doi: 10.1371/journal.pone.0104764 (PMC4128720; doi:10.1371/journal.pone.0104764)

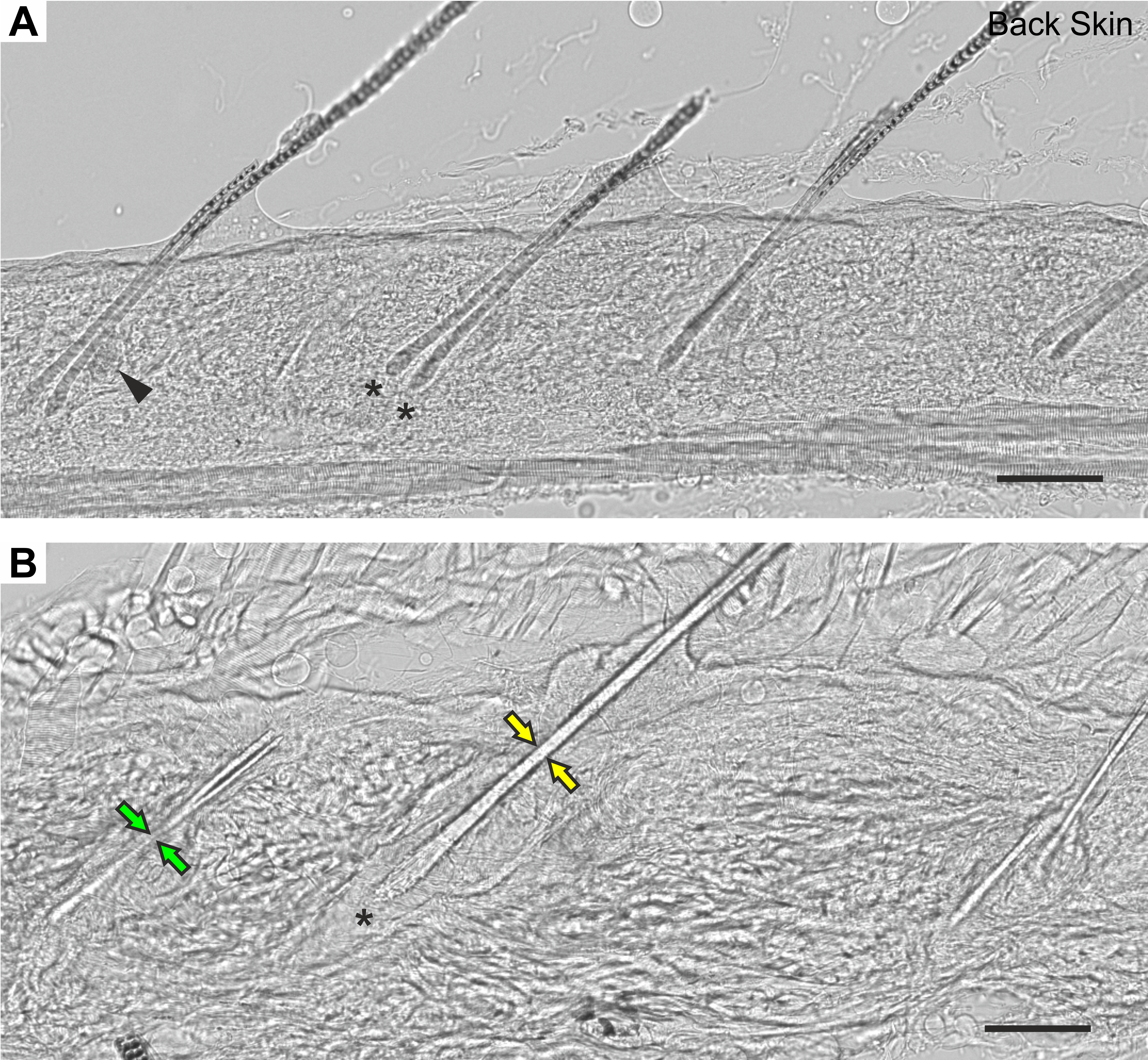

Supplement: Figure S1 — Bright-field images from wild-type mouse back skin. (A) The image shows several small caliber hair follicles representative of the type used in the analysis of TH+ LLE innervation. The black arrowhead points to a sebaceous gland next to a hair follicle. Note that the small hair follicles generally form closely associated pairs (asterisks). (B) The image shows several small and one large caliber hair follicle. The large hair follicle (asterisk) is representative of the type used in NFH+ LLE innervation analysis. Note the considerably larger diameter and singular hair shaft (yellow arrows) in the large follicle compared to a small hair follicle (green arrows). Scale bars: 100 µm. (TIF) [file pone.0104764.s001.tif]

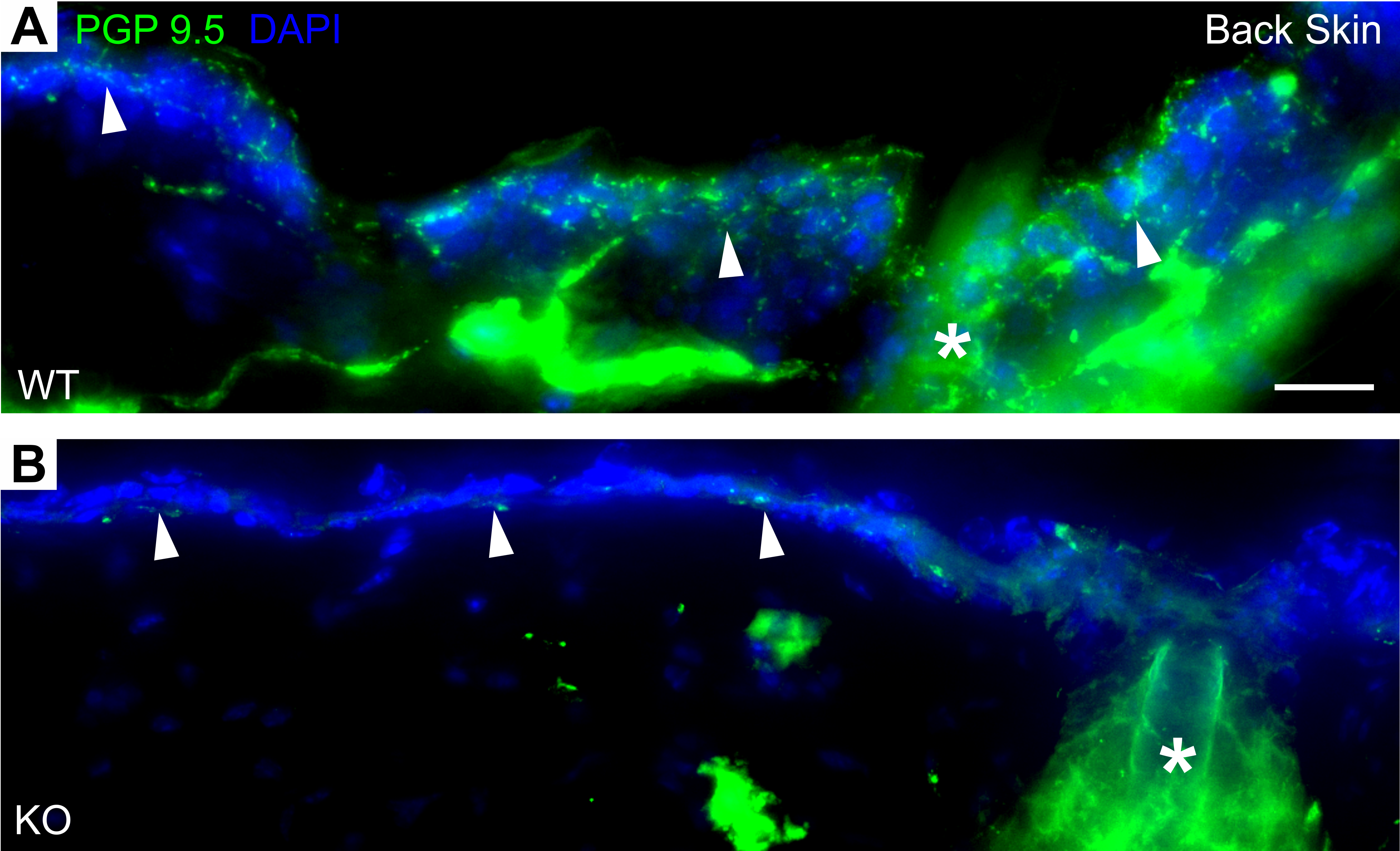

Supplement: Figure S2 — Epidermal innervation in GFRα2-KO mouse back skin is reduced. (A) WT mouse back skin epidermis is densely innervated by PGP9.5+ nerve fibers. (B) Epidermal innervation is sparse in KO back skin epidermis. Arrowheads in (A) and (B) exemplify the difference in innervation density between the genotypes. Asterisks mark large caliber hair shafts. The images are maximum projections from conventional microscopic stacks. Scale bar: 25 µm. (TIF) [file pone.0104764.s002.tif]

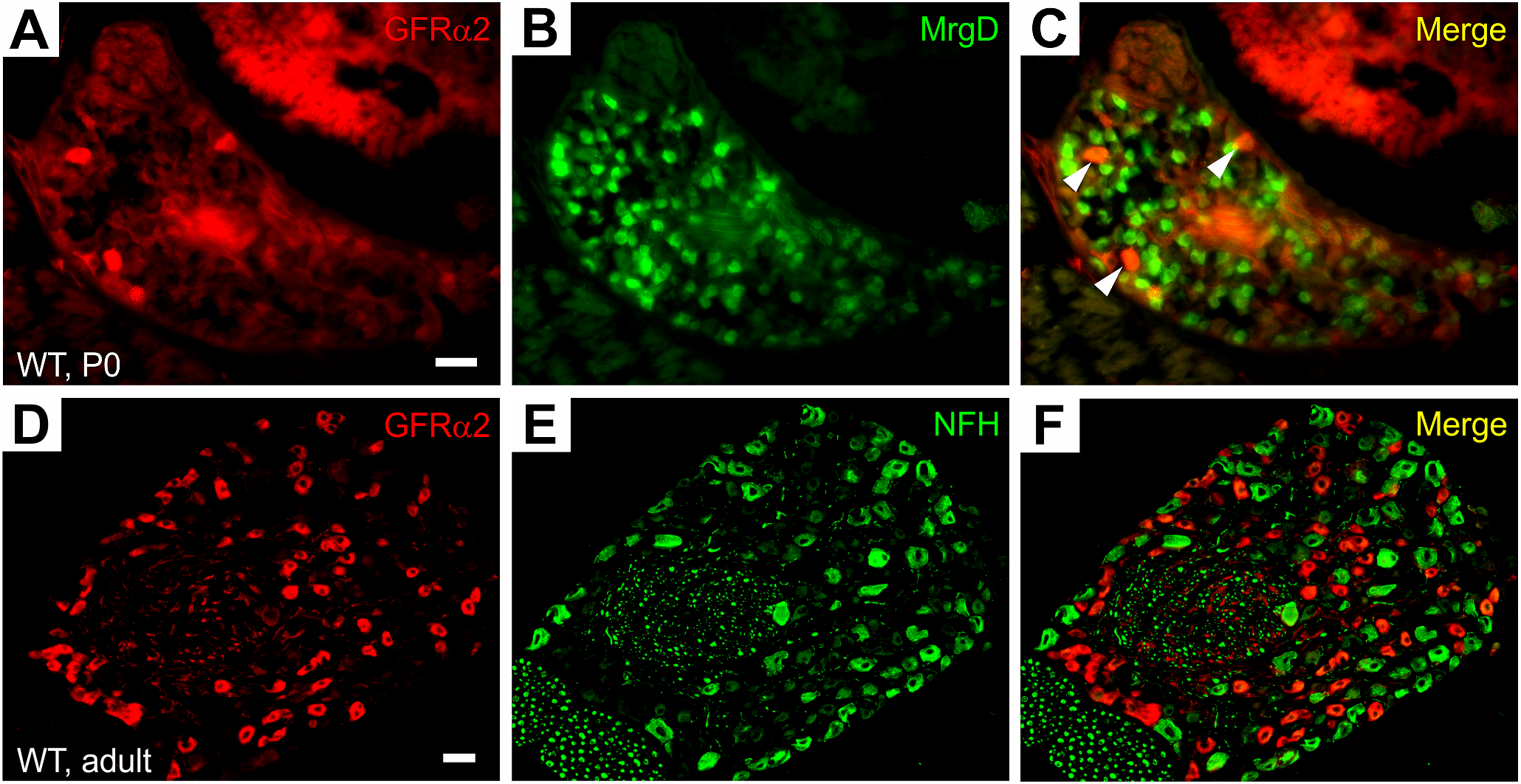

Supplement: Figure S3 — GFRα2 is not detectable in large NFH+ DRG neurons in adult mice. (A–C) Conventional microscopic images demonstrating that GFRα2 expression is restricted to a minor population of large DRG neurons at P0 (A), while MrgD is expressed in a major population of smaller neurons (B). (C) GFRα2 and MrgD do not colocalize in newborn mouse DRGs (arrowheads point to single-positive GFRα2+ neurons). (D–F) Maximal projection images showing that GFRα2 is abundantly expressed in small to medium diameter neurons in adult mouse thoracic DRGs (D) and that NFH is expressed in many medium to large sized DRG neurons (E). (F) GFRα2 and NFH do not colocalize in adult mouse DRGs. Scale bars: 50 µm. (TIF) [file pone.0104764.s003.tif]

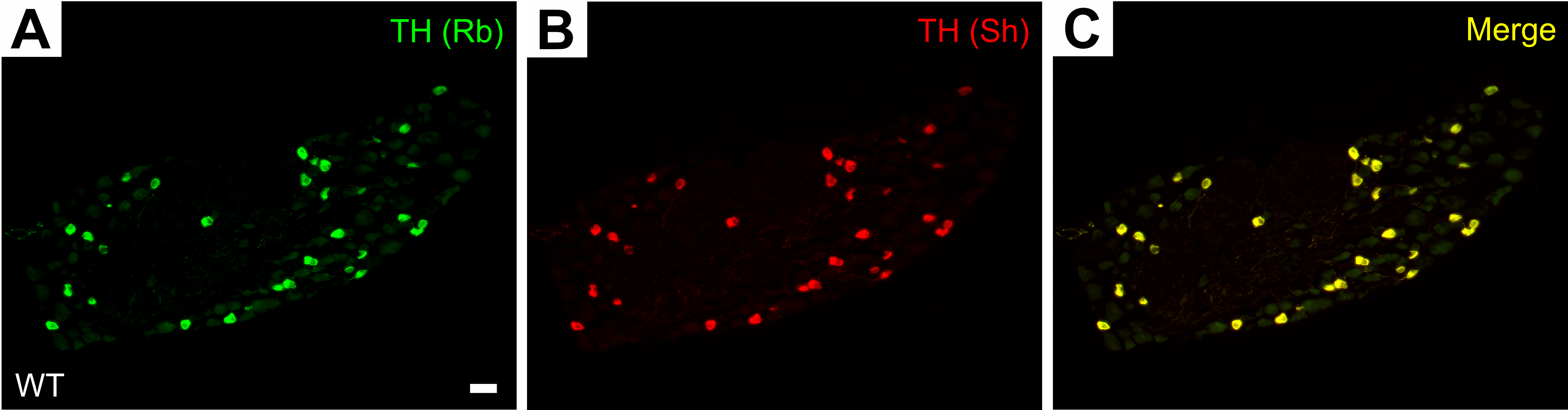

Supplement: Figure S4 — Two different anti-TH antibodies stain the same population of DRG neurons. (A, B) Representative images from a WT mouse thoracic DRG section stained with (A) rabbit anti-TH and (B) sheep anti-TH antibody. (C) The merged image shows that the two antibodies label the same cell population. This virtually 100% colocalization was confirmed by analysis of 356 TH+ cells (in five DRGs from two animals). Scale bar: 50 µm. (TIF) [file pone.0104764.s004.tif]
