# Supplementary material for: Different Requirements for GFRα2-Signaling in Three Populations of Cutaneous Sensory Neurons
Source: PLoS One. 2014 Aug 11;9(8):e104764. doi: 10.1371/journal.pone.0104764 (PMC4128720; doi:10.1371/journal.pone.0104764)
Supplement: Table S1 — Primary antibodies used in immunohistochemistry. (PDF) [file pone.0104764.s005.pdf]

Table S1. Primary antibodies used in immunohistochemistry.

Antibody	Host	Supplier	Antibody ID	Immunogen	Dilution
anti-GFRα2	Goat	R&D Systems	AF429	NS0-derived recombinant mouse GFRα2 extracellular domain [1]	1:200
anti-Ret	Goat	Neuromics	GT15002	Recombinant mouse Ret extracellular domain [2]	1:100
anti-TH	Rabbit	Millipore	AB152 (LOT:0612047287)	Denatured rat tyrosine hydroxylase [3]	1:400
anti-TH	Sheep	Millipore	AB1542	Native rat tyrosine hydroxylase [4]	1:400
anti-NFH	Rabbit	Millipore	AB1989	Recombinant fusion protein containing the extreme C-terminus of rat NF-H [5]	1:1000
anti-GFP	Rabbit	Molecular Probes	A-6455	GFP isolated directly from the jellyfish *Aequorea victoria* [6]	1:1000
anti-TrkB	Goat	R&D Systems	AF1494	NS0-derived recombinant mouse TrkB [7]	1:1000
anti-PGP9.5	Rabbit	Millipore	AB1761	Recombinant human Protein Gene Product 9.5 (PGP9.5, Ubiquitin C-terminal Hydrolase, UCH-L1) [8]	1:400

References:

[1] http://www.rndsystems.com/pdf/af429.pdf

[2] http://www.neuromics.com/site/special/A8x475x8x1.pdf

[3] http://www.millipore.com/catalogue/item/ab152

[4] http://www.millipore.com/catalogue/item/ab1542

[5] http://www.millipore.com/catalogue/item/ab1989

[6] http://www.lifetechnologies.com/order/catalog/product/A6455

[7] http://www.rndsystems.com/pdf/af1494.pdf

[8] http://www.emdmillipore.com/FI/en/product/Anti-Protein-Gene-Product-9.5-Antibody,MM_NF-AB1761
